# 1945. Antibacterial activity of cefepime/enmetazobactam and comparator agents against U.S. Enterobacterales clinical isolates collected from 2019-2021

**DOI:** 10.1093/ofid/ofad500.099

**Published:** 2023-11-27

**Authors:** Adam Belley, Nimmi Kothari, Federica Monti, Stephen Hawser

**Affiliations:** Allecra Therapeutics SAS, Beaconsfield, Quebec, Canada; IHMA, Monthey, Valais, Switzerland; IHMA, Monthey, Valais, Switzerland; IHMA Europe, Monthey, Valais, Switzerland

## Abstract

**Background:**

The investigational β-lactam/β-lactamase inhibitor combination of cefepime/enmetazobactam (FPE) met criteria for non-inferiority and superiority compared to piperacillin-tazobactam (PTZ) in a phase 3 clinical trial of adult patients with complicated urinary tract infections/acute pyelonephritis. FPE has demonstrated potent *in vitro* antibacterial activity against Gram-negative pathogens expressing extended spectrum β-lactamases (ESBL), the leading cause of resistance to 3^rd^-generation cephalosporins (3GC). In this study, the *in vitro* activity of FPE and comparator agents was assessed against Gram-negative clinical isolates obtained from the U.S. between 2019 and 2021.

**Methods:**

Broth microdilution minimum inhibitory concentrations (MIC) were determined for 2,241 Enterobacterales clinical isolates following CLSI guidelines. For determination of phenotypic resistance to 3GC, isolates resistant to both ceftriaxone and ceftazidime but susceptible to the carbapenem meropenem (MEM) were selected. ESBL genotyping was performed using a multiplex PCR method. Cefepime breakpoints of susceptible (S) ≤2 µg/ml and susceptible-dose dependent (SDD) ≤8 µg/ml were used as provisional breakpoints for FPE.

**Results:**

FPE demonstrated potent *in vitro* activity against the surveillance isolates obtained from US medical centers, with an MIC_90_ of 0.12 µg/ml that was comparable to those of MEM (0.06 µg/ml) and ceftazidime/avibactam (CZA; 0.25 µg/ml). When applying cefepime breakpoints, susceptibility to FPE was ≥98.4 µg/ml whereas susceptibility to 3GCs and PTZ was ≤85.8%. Against Enterobacterales with either an ESBL genotype or exhibiting a 3GC-resistant phenotype, FPE retained potent *in vitro* activity (MIC_90_ = 0.5 and 1 µg/ml) and provisional susceptibility was ≥95.6%. In contrast, susceptibility to 3GCs and PTZ ranged from 0.0 to 65.9%.

Summary of the in vitro activity of cefepime/enmetazobactam and comparator agents against US Enterobacterales clinical isolates collected from 2019-2021

**Figure:**
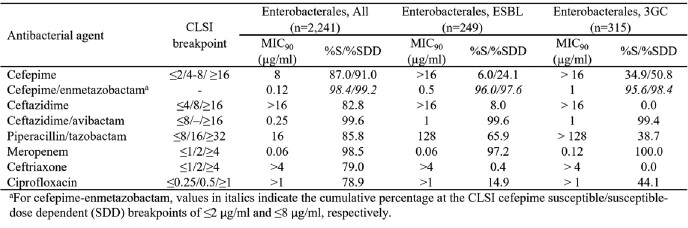

**Conclusion:**

These results demonstrate that diminished susceptibility to 3GCs and piperacillin/tazobactam has compromised their reliability as empiric therapies. Cefepime/enmetazobactam demonstrates potent *in vitro* activity comparable to meropenem against ESBL-producing Enterobacterales and is intended as empiric therapy in settings where ESBL are endemic.

**Disclosures:**

**Adam Belley, PhD**, Allecra Therapeutics SAS: Advisor/Consultant **Nimmi Kothari, PhD**, Allecra: Allecra (study funding)|Innoviva Specialty Therapeutics, Inc.: Honoraria|Roche: Honoraria|Roche: This project has been funded by BARDA (HHSO100201600038C). **Federica Monti, PhD**, Allecra: Study funded|Innoviva Specialty Therapeutics, Inc.: Honoraria **Stephen Hawser, PhD**, Allecra: study funding|Innoviva Specialty Therapeutics, Inc.: Honoraria|Roche: Honoraria|Roche: This project has been funded by BARDA (HHSO100201600038C).

